# Characterisation of the Major Extracellular Proteases of *Stenotrophomonas maltophilia* and Their Effects on Pulmonary Antiproteases

**DOI:** 10.3390/pathogens8030092

**Published:** 2019-06-28

**Authors:** Kevin Molloy, Stephen G. Smith, Gerard Cagney, Eugene T. Dillon, Catherine M. Greene, Noel G. McElvaney

**Affiliations:** 1 Department of Medicine, Royal College of Surgeons in Ireland, Beaumont Hospital, D02 YN77 Dublin, Ireland; 2 Department of Clinical Microbiology, School of Medicine, Trinity College Dublin, D02 PN40 Dublin, Ireland; 3 School of Biomolecular and Biomedical Science, University College Dublin, D04 V1W8 Dublin, Ireland; 4 Department of Clinical Microbiology, Royal College of Surgeons in Ireland, Beaumont Hospital, D02 YN77 Dublin, Ireland

**Keywords:** serine protease, cystic fibrosis, alpa-1 antitrypsin, secretory leucoprotease, elafin

## Abstract

*Stenotrophomonas maltophilia* is an emerging global opportunistic pathogen that has been appearing with increasing prevalence in cystic fibrosis (CF). A secreted protease from *S. maltophilia* has been reported as its chief potential virulence factor. Here, using the reference clinical strain *S. maltophilia* K279a, the major secreted proteases were identified. Protein biochemistry and mass spectrometry were carried out on K279a culture supernatant. The effect of K279a culture supernatant on cleavage and anti-neutrophil elastase activity of the three majors pulmonary antiproteases was quantified. A deletion mutant of *S. maltophilia* lacking expression of a protease was constructed. The serine proteases StmPR1, StmPR2 and StmPR3, in addition to chitinase A and an outer membrane esterase were identified in culture supernatants. Protease activity was incompletely abrogated in a K279a-ΔStmPR1: Erm mutant. Wild type K279a culture supernatant degraded alpha-1 antitrypsin (AAT), secretory leucoprotease inhibitor (SLPI) and elafin, important components of the lung’s innate immune defences. Meanwhile SLPI and elafin, but not AAT, retained their ability to inhibit neutrophil elastase. StmPR3 together with StmPR1 and StmPR2, is likely to contribute to protease-mediated innate immune dysfunction in CF.

## 1. Introduction

Bacterial proteases have important pathological consequences in cystic fibrosis (CF) lung disease. The role of some, in particular proteases produced by *P. aeruginosa*, an opportunistic CF lung pathogen, have been investigated [1,2]. Less is known regarding the role of proteases in the pathogenesis of *Stenotrophomonas maltophilia* lung disease in CF. *S. maltophilia* is commonly present in the CF respiratory tract [3,4]. The pathogenic role of this organism in CF is controversial with recent evidence indicating that colonisation is associated with an almost threefold increased risk of death or lung transplantation [5].

*S. maltophilia* produces both a major (StmPR1) and a minor (StmPR2) [6,7,8] extracellular protease. The *stmPR1* gene encodes a 63 kDa precursor which is post-translationally modified to a functionally active 47 kDa subtilisin-like serine protease. StmPR1 is an alkaline protease with an optimal pH of 9.0 and its activity can be inhibited in the presence of antipain, chymostatin, and phenylmethylsulfonyl fluoride (PMSF) [6,7,8]. Significant inter strain differences in StmPR1 expression exist [9]. Two distinct allelic variants of *stmPR1* have been identified; the original one first identified by Windhorst et al. [6] and the second in K279a, the reference clinical strain [7]. The genome of *S. maltophilia* K279a has been fully sequenced [10] and is now the reference clinical strain of choice for studying *S. maltophilia* virulence related mechanisms, and is used throughout this work. 

StmPr2 is expressed in K279a [7,8] Using both clinical and an environmental strains of *S. maltophilia*, further serine proteases from *S. maltophilia* have been identified, termed StmPR3 and StmPR4 [8,11]. An ATP-dependent metalloproteinase (FtsH) [11] and an esterase have also been described [9].

Extracellular protease production from *S. maltophilia* may have important pathogenic consequences in persons colonised by the organism, being responsible for degradation of components of the extracellular matrix, immunoglobulin G [6] and for destructive effects on airway epithelial cells [12]. StmPR1 was a more important virulence determinant than StmPR2 in a toxicity model in *Galleria mellonella* larvae [9]. Indeed, a series of studies has reported genomic and biochemical information on StmPR1, 2 and 3 under various bacterial growth conditions, protease mutant construction and demonstration that the proteases are secreted via a type II secretion system [7,8]. In addition, evidence exists of serine proteolytic and caseinolytic activity by *S. maltophilia*, that is sensitive to PMSF and chymostatin, and can degrade host extracellular matrix proteins and IL-8, induce human lung epithelial cell rounding, detachment and, death, and activate PAR receptors [7,8]. Production of an extracellular protease has been purported to be responsible for the pathological effects seen in cases of lethal haemorrhage in *S. maltophilia* pulmonary infection [13] and could also be responsible for virulence in CF lung disease. 

The CF lung is an intense pro-inflammatory environment, the hallmark of which is neutrophil dominated inflammation and high amounts of the serine protease, neutrophil elastase (NE) [14]. Three important pulmonary serine anti-proteases are alpha-1 antitrypsin (AAT), secretory leucoprotease inhibitor (SLPI) and elafin [15]. These have important beneficial effects within the lung and their dysregulated activity can impact adversely on the inflammatory process [14,15]. Bacterial proteases have the potential to destroy host tissues as well as to destroy proteins that are important components of the innate immune defences [16,17,18,19]. For example, *Pseudomonas* proteases can cleave AAT [20], SLPI [21] and elafin [1]. The direct effect of *S. maltophilia* proteases on pulmonary anti-proteases has not been investigated.

AAT, a 52 kDa glycoprotein is the canonical serine protease inhibitor within the lung and is the most important inhibitor of NE. In the CF lung, the inhibitory activity of AAT is overwhelmed by an excess of harmful proteases, in particular neutrophil elastase (NE) but also cathepsin L [22] and *Pseudomonas* elastase. SLPI, an 11.7 kDa cationic protein, is a potent inhibitor of NE and can also inhibit cathepsin G [23]. It also has antibacterial, antiviral and anti-inflammatory properties [24,25,26,27,28]. SLPI is susceptible to cleavage by cathepsins B, L and S [29]. Elafin is expressed in the lung and other sites [30,31]. It possesses anti-microbial and anti-inflammatory activities [32,33,34] and is susceptible to NE-mediated cleavage in the lungs of patients colonised with *P. aeruginosa* [35]. 

In the CF lung, the important anti-inflammatory, anti-microbial and anti-protease effects of AAT, SLPI and elafin are lost but the role of *S. maltophilia* in mediating these effects has not yet been determined. Here we elucidate its major extracellular protease(s) using K279a as the reference clinical strain of *S. maltophilia* and investigate the ability of K279a culture supernatant to degrade AAT, SLPI and elafin and determine the functional consequences on their ability to inhibit neutrophil elastase. In addition, we generate a mutant of *S. maltophilia* defective in StmPR1.

## 2. Results

### 2.1. Optimal Conditions for K279a Extracellular Protease Production

**Figure 1:**
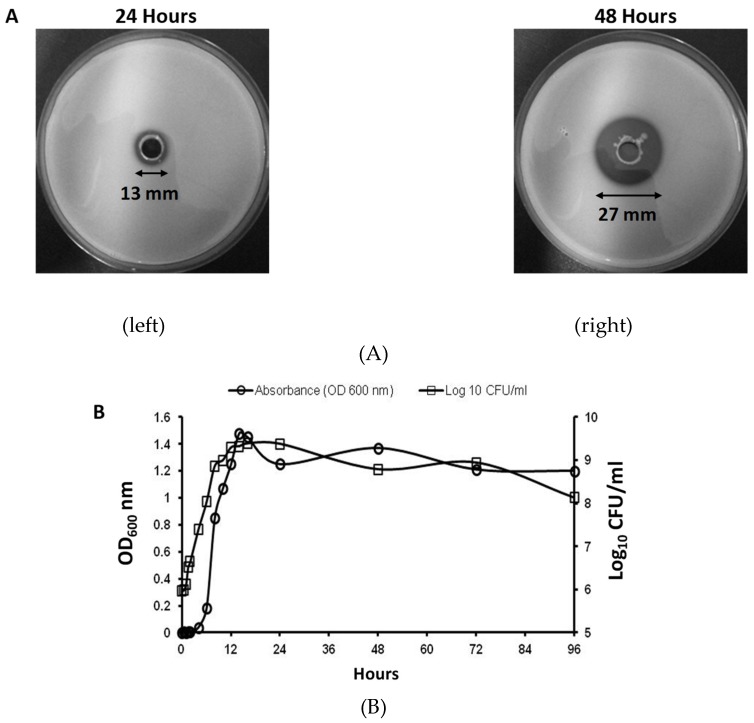
Initial screening for protease activity using LB agar 2% milk plates and K279a growth curve. (**A**). Following overnight culture, 100 μL of K279a (grown in LBB at 37°C and 200 rpm) was inoculated in a centrally cored 0.8 cm well in a LB agar plate containing 2% skim milk. Plates were photographed at 24 h (left) and 48 h (right) following incubation and examined for zones of hydrolysis (indicated by central clearing of halos). (**B**) A K279a growth curve was constructed following inoculation of 10 μL of an overnight culture of K279a (grown in LBB at 37 °C and 200 rpm) in fresh LBB. Absorbance at 600 nm (OD_600_) and a CFU/mL count was calculated every 2 h for the first 16 h and every 24 h thereafter for a total duration of 96 h. Results are expressed as OD_600_ nm (right y-axis) and Log_10_ CFU/mL (left y-axis).

Initially to screen for phenotypic expression of proteolytic activity in K279a, the ability of K279a to form clear zones when grown on indicator LB agar medium containing 2% skimmed milk was assessed, similar to Karaba et al. [12]. Halo formation was caused by hydrolysis of the milk protein casein. K279a formed clear halos at 24 h (13 mm) and 48 h (27 mm), respectively (Figure 1A) corresponding to the early to mid-stationary phase of growth (Figure 1B).

**Figure 2:**
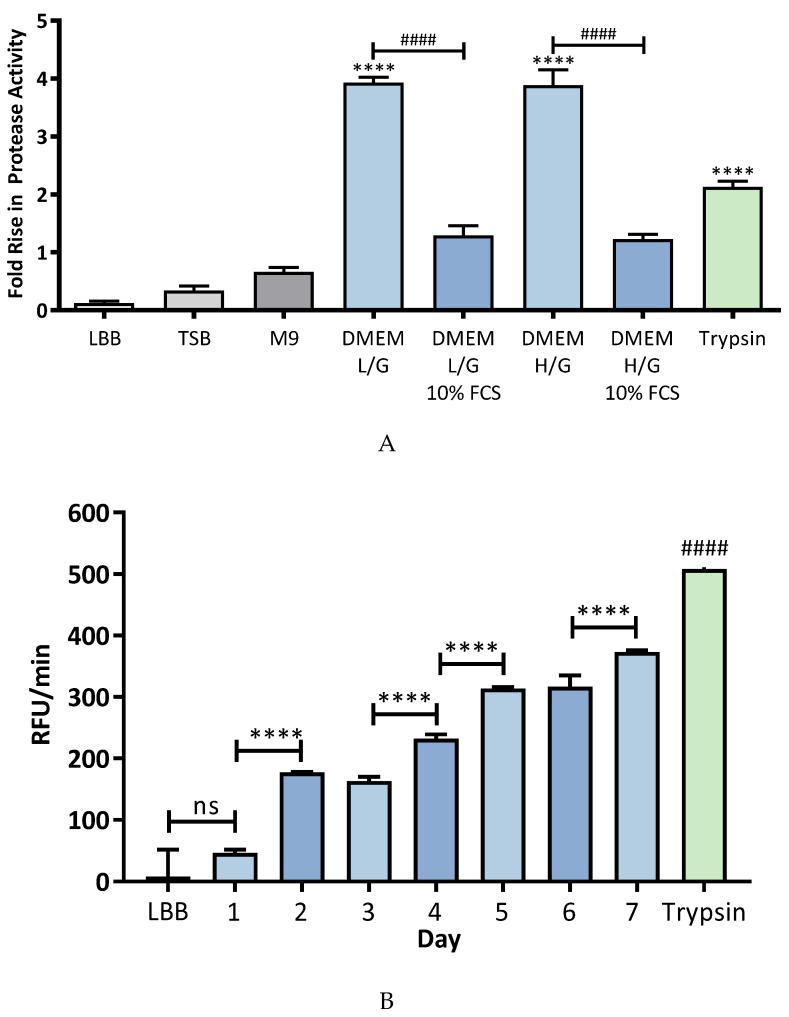
Effect of culture medium and time on K279a protease activity. (**A**) K279a culture supernatant was obtained following inoculation of 10 μL of an overnight culture of K279a (grown in LBB at 37 °C and 200 rpm) in the following test media: LBB, TSB, M9 minimal media, DMEM-L/G ± 10% v/v FCS, DMEM-H/G ± v/v 10%FCS. Each culture was incubated for 48 h at 37 °C and 200 rpm. Protease activity in culture supernatants was measured using the Sensolyte Red Protease assay kit (Anaspec). K279a culture supernatant obtained from LBB at 48 h and trypsin (12.5 mU/μL) were used as positive controls. Results are expressed as fold rise in protease activity relative to broth control and are representative of three independent experiments. All measurements are represented as means ± SEM from biological replicates. One-way-ANOVA followed by Tukey post hoc test for multiple comparisons; ns (non significant) LBB vs day 1, ****(*p* ≤ 0.0001) protease activity in test media vs LBB K279a culture supernatant, #### (*p* ≤ 0.0001) protease activity in DMEM L/G or H/G ± 10% v/v FCS. (**B**) K279a culture supernatant from LBB cultures following inoculation of 10 μL of an overnight culture and prepared on days 1-7 by centrifuging at 1000 rcf for 5 min to pellet the cells followed by filter sterilisation through a 0.22 μm Millex syringe filter. Protease activity was measured using the Sensolyte Red Protease assay kit (Anaspec). LBB and trypsin (12.5 mU/μL) were used as negative and positive controls. Results are representative of three independent experiments and are represented as means ± SEM from biological replicates. One-way-ANOVA followed by Tukey post hoc test for multiple comparisons; ns (non significant) LBB vs day 1; ****(*p* ≤ 0.0001) protease activity of K279a culture supernatant per days of culture 1-2, 3-4, 4-5 and 6-7; #### (*p* ≤ 0.0001), LBB vs trypsin control.

Having determined that K279a is proteolytically active at 24 and 48 h on solid medium protease, production in broth liquid culture (LBB) was confirmed using the Sensolyte Red Protease assay. DuMont et al. previously demonstrated the enzymatic activity StmPR proteins in buffered yeast extract [7,8]. In LBB, significantly more protease activity was evident following 48 h of culture compared with 24 h and no significant difference was observed between the 24-h time-point and uninoculated control (Figure 2B). A subsequent increase in activity was observed between days 3–4, 4–5 and 6–7. However, this later increase in protease activity likely represented a decline phase in bacterial growth and was therefore not investigated any further. For subsequent experiments all culture supernatants were sampled after 48 h of culture. 

In order to determine a suitable nutrient medium for the maximal extracellular protease activity, K279a culture supernatant was prepared using a selection of different culture media including LBB, trypticase soy broth (TSB), M9 minimal medium and the mammalian cell culture medium Dulbecco’s modified essential medium (DMEM) [high (25 mM) and low glucose (5.6 mM) versions] with and without 10% foetal calf serum (Figure 2A). Growth in DMEM resulted in the highest fold (3.933 ± 0.092) increase in protease activity compared with LBB (*p* < 0.0001). The concentration of glucose had no effect on protease, being similar in DMEM with a high glucose concentration (25 mM) and a low glucose concentration (5.6 mM). The presence of foetal calf serum (FCS) reduced K279a protease activity in DMEM L/G (1.294 ± 0.1684) and H/G (1.227 ± 0.08744) likely due to the potential presence of protease inhibitors such as AAT and alpha-2 macroglobulin in FCS. As no significant difference between low glucose and high glucose versions of DMEM was identified, DMEM with the lower glucose concentration (5.6 mM) was used for all subsequent experiments and will be referred to as DMEM from this point forward. Indeed, this concentration is of the order observed in the CF lung [36,37].

Concurrent with this experiment bacterial growth characteristics were assessed by calculating log CFU/mL of K279a in each of the respective growth media. On close examination of Figure 2A no significant difference in K279a protease activity was identified between rich culture media (LBB and TSB) and the minimal M9 growth medium following correction for multiple comparisons using a Tukey post-hoc test. The fold rise in protease activity relative to broth control between LBB, TSB and M9 growth medium was 0.124, 0.3444 and 0.6642, respectively. ANOVA analysis of these three media alone demonstrated a statistically significant difference between LBB and M9 (*p* = 0.002) and TSB and M9 (*p* = 0.03) after correction for multiple comparisons. Examination of growth characteristics of K279a in these respective media showed that K279a grew robustly in LBB, TSB and DMEM (low/high glucose) with or without 10% foetal calf serum (data not shown). In contrast, growth of K279a in M9 minimal medium is 75% less efficient than LBB and TSB and 66% less efficient to the DMEM variants (data not shown). Thus, even in suboptimal growth conditions (as with M9 medium) K279a proteases are more readily produced than in the nutrient rich media such as LBB and TSB but not quite as avidly as with DMEM, which we deemed to have the optimal *in vitro* conditions for protease induction.

### 2.2. The Major Secreted Extracellular Protease of K279a Exhibits Serine Protease Activity

**Figure 3:**
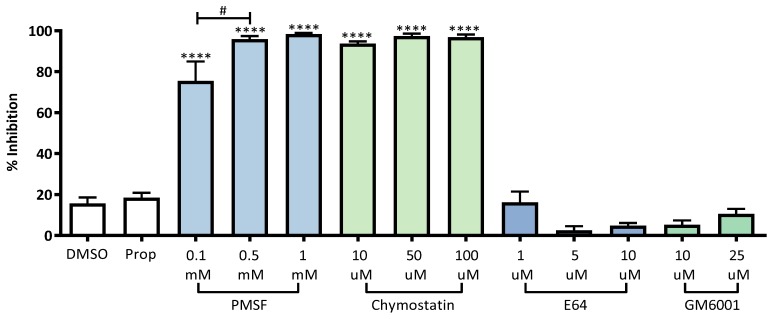
K279a extracellular proteases are inhibited by PMSF and chymostatin. K279a culture supernatant was treated with PMSF (0.1 mM, 0.5 mM and 1 mM), chymostatin (10 µM, 50 µM and 100 µM), E64 (1 µM, 5 µM and 10 µM) or GM6001 (10 µMa 50 µM) and protease activity was measured using the Sensolyte Red Protease assay relative to their vehicle controls (PMSF = propanol, Chymostatin, E64, GM6001 = DMSO). Relative % inhibition was calculated using the reaction velocity of the untreated vehicle (V0) control and that of the tested protease inhibitors (V1) using the equation 100 × (1 – V1/V0). Velocity was calculated based on the slope of the line using the linear regression equation: y = mx + c (m denoting the slope) on the steepest portion of the kinetic curve. All results are representative of three independent experiments and are represented as means ± SEM from biological replicates. One-way ANOVA followed by Tukey post hoc test; **** *p* ≤ 0.0001: Inhibitor vs. vehicle control. Student *t*-test; # *p* ≤ 0.05: PMSF 0.1 mM vs 0.5 mM. Abbreviations: DMSO, Dimethyl sulfoxide; Prop, 2-propanol; PMSF, phenylmethylsulfonyl fluoride.

Having optimised growth conditions for maximal production of protease activity, we next confirmed the classes of proteases secreted by K279a. To this end the inhibitory capacity of a variety of protease inhibitors was assessed including PMSF (phenylmethylsulfonyl fluoride), an irreversible inhibitor of serine proteases and chymostatin, a bioactive peptide with selectivity for chymotryptase-like serine proteases identified previously as a potent inhibitor of StmPR1, the major secreted extracellular protease by *S. maltophilia* [6,7,8]. Other protease inhibitors assessed included E64, an irreversible inhibitor of cysteine peptidases and GM6001, a broad-spectrum metalloproteinase inhibitor. Figure 3 shows that PMSF and chymostatin, at all concentrations tested, significantly inhibited K279a extracellular protease activity (*p* ≤ 0.0001) relative to their vehicle controls propanol and DMSO, respectively. No inhibition by GM6001 and E64 was evident. Therefore, our studies (though performed in different media) support other published works reporting that the major secreted extracellular proteases belong to the serine protease class of enzymes [6,7,8].

### 2.3. Screening K279a for Extracellular Serine Proteases

**Figure 4:**
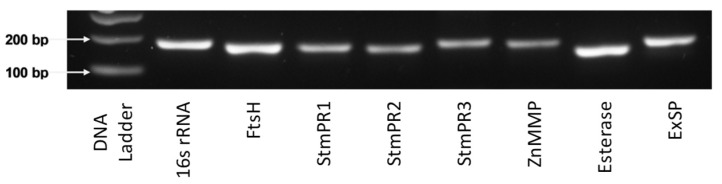
Candidate protease genes identified from the genome of K279a by PCR. Genomic DNA was extracted from overnight cultures of K279a (grown in LBB at 37 °C with agitation at 200 rpm) using the Fast DNA spin kit (MP Bio). DNA concentration was quantified using the NanoDrop 8000 (Thermo Scientific) and a final concentration of 100 ng was used for PCR with SYBR Green I Master on the Roche LC480 lightcycler. PCR products were electrophoresed on 1.5% agarose gel electrophoretograms using a 100 bp (New England Biolabs) DNA ladder. Abbreviations: 16s rRNA = 16s ribosomal RNA; FtsH = ATP dependent metalloproteinase; StmPR1, 2 and 3 = Subtilisin like serine proteases; ZnMMP = Zinc metalloproteinase; ExSP = Extracellular serine protease.

The *S. maltophilia* K279a genome has been sequenced (GenBank: AM743169.1). It encodes two metalloproteinase genes, one esterase gene and four serine protease genes, namely, FtsH, ZnMMP, esterase, StmPR1, StmPR2, StmPR3 and ExSP [7,8,9,10,11]. The presence of these protease genes on the genome of K279a was validated by PCR using specific primers to each protease gene of interest on K279a genomic DNA. A 1.5% agarose gel verified single products of the predicted size, indicating the carriage of all of protease genes of interest in the genome of K279a (Figure 4).

Given that a serine protease(s) was responsible for almost all extracellular proteolytic activity, we focused on candidate proteases with serine-type endopeptidase activity, namely StmPR1, StmPR2, StmPR3 and ExSP. These belong to the peptidase S8 or subtilase family of serine proteases as classified by the MEROPS peptidase database (www. merops.sanger.ac.uk) with an Asp, His and Ser catalytic triad. Table 1 summarises the features of each of the candidate extracellular serine proteases including UniProt ID, gene name, sequence length and predicted molecular weights. The predicted isoelectric points (PI) were obtained from ExPASy (http://web.expasy.org/compute_pi/). 

**Figure 5:**
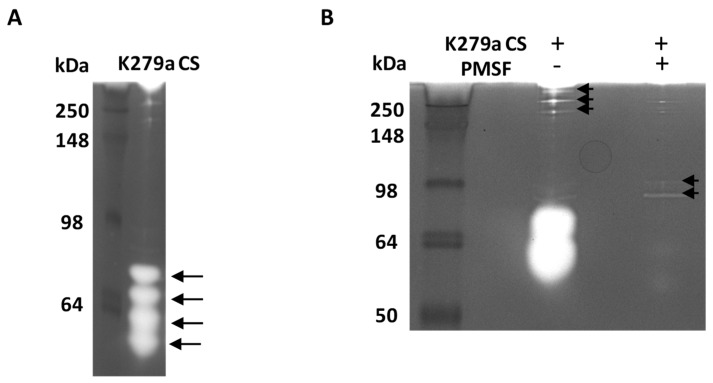
Gelatin zymography of K279a culture supernatant. A 1:10 dilution of K279a culture supernatant was either untreated or pre-incubated with 1 mM of PMSF before being mixed in a 1:1 ratio with non-reducing sample buffer. 10 μL of sample was loaded per well and subjected to electrophoresis for (**A**) 4 h or (**B**) 1.5 h on a 12.5% gelatin zymogram. Zones of gelatinolytic activity are indicated by the black arrows.

To visualise the proteolytic activity of K279a secreted proteases, gelatin zymography was utilised to identify bands with gelatinolytic activity and approximate their molecular weight. Gelatin zymography of K279a culture supernatant (diluted 1:10 in DMEM) confirmed that the major secreted extracellular proteases from K279a were serine proteases, being inhibited by the serine protease inhibitor PMSF. Four major bands (Figure 5A) and five minor bands (Figure 5B) were identified. The activity of all the major bands (data not shown) and three of the minor bands (Figure 5B) was abolished in the presence of PMSF indicating the presence of high molecular (approximately 250 kDa) and lower molecular weight (approximate MW range 55–80 kDa) secreted serine proteases. PMSF had no effect on the activities of two of the minor bands with intermediate molecular weight (~100 kDa) and indicated they belonged to a different class of proteases.

### 2.4. Identification of the Major Secreted Proteases by Mass Spectrometry 

**Figure 6:**
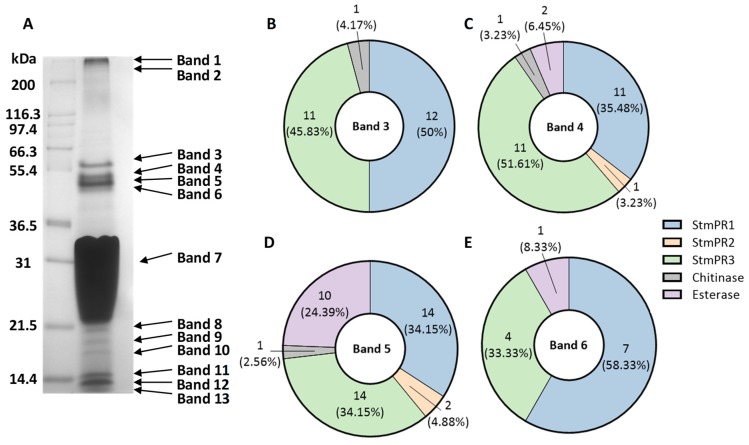
Identification of the major extracellular serine proteases by in-gel digestion and mass spectrometry analysis. (**A**) K279a culture supernatant proteins were filtered and concentrated and loaded onto a 12.5% polyacrylamide gel, subjected to SDS-PAGE, and stained with colloidal Coomassie blue silver stain. The relative migrations of molecular mass protein standards (lane 1; in kDa) are indicated on the left and K279a culture supernatant proteins to the right. Respective bands are indicated by the arrows. (**B**–**E**) Spectral counts of peptides identified by LC-MS/MS from in gel-digestion of proteases in bands 3-6. Each protease is represented by a unique colour. The spectral count is indicated by a whole number and denoted below is the percentage spectral count of each protease per band.

As a means of identifying the proteases secreted by K279a, culture supernatant concentrates from broth cultures of K279a were examined by SDS-PAGE and Coomassie blue silver staining (Figure 6A). This revealed clustering of four bands adjacent to the 55.4 kDa molecular weight standard (bands 3–6). The most intensely staining band had an approximate molecular weight of 47 kDa which may correspond to mature StmPR1 (band 5) as described by Windhorst et al. [6]. Three additional bands, (bands 3, 4, 6) may contain the additional serine proteases of interest while the two high molecular weight bands (bands 1, 2) may contain proteases representative of the high molecular weight areas of protease activity that were observed by gelatin zymography (Figure 5A). 

Given that the most intense area of protease activity on zymography was located adjacent to the 64 kDa molecular weight standard, we hypothesised that the most intensely staining bands on SDS-PAGE and Coomassie staining adjacent to the 55.4 kDa molecular weight standard contained the proteases of interest. These included StmPR1, StmPR2 and StmPR3 but not ExSP with unprocessed molecular weights of 63 kDa, 58 kDa and 61 kDa respectively. Four fractions of gel bands were excised (bands 3–6) and in-gel digestion was performed on each fraction. After in-gel digestion of proteins, LC-MS/MS was used to analyse the extracted peptides for protein identification. All three proteases of interest were identified in addition to chitinase A (UniProt ID: B2FMT1) and an outer membrane esterase (UniProt ID: B2FSC8) (Table 2). 

Relative protein quantitative levels were determined by spectral counting. StmPR1 and StmPR3 were the most abundant subtilisins (serine protease) identified and were detected in all four digested bands. StmPR2, the so-called minor extracellular protease and chitinase A, a glycoside hydrolase with anti-fungal properties were present in bands 4 and band 5 but were less abundant than StmPR1 and StmPR3 [38]. The outer membrane esterase was more readily represented in band 5 but was also detected in bands 4 and 6 (Figure 6B–E). These results indicate that StmPR1 is not the sole major serine protease secreted by *S. maltophilia* but that StmP3 may also be a major extracellular protease of K279a. 

### 2.5. Incomplete Attenuation of Protease Activity in a K279a StmPR1 Mutant 

**Figure 7:**
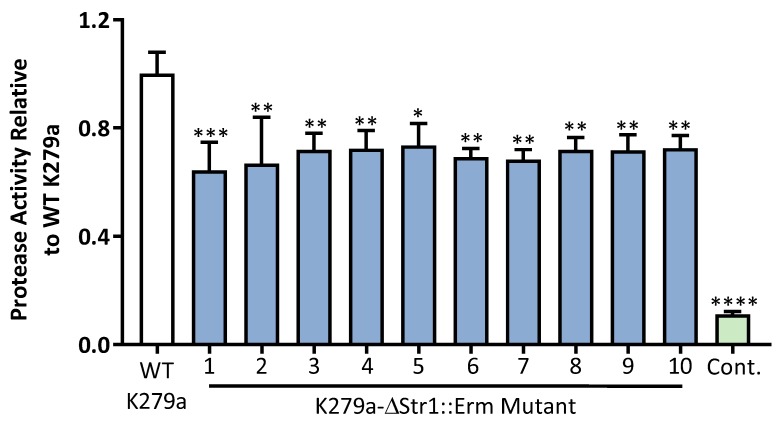
Extracellular protease activity is incompletely attenuated in selected K279a-ΔStr1: Erm mutants. Culture supernatants were obtained following inoculation of a single colony of WT K279a and 10 selected K279a-ΔStr1: Erm. Each culture was incubated for 48 h at 37 °C and 200 rpm and bacterial culture supernatant was obtained as previously described. Protease activity was measured using the Sensolyte Red Protease assay kit (Anaspec). WT K279a culture supernatant was used as a positive control while DMEM culture medium was used as a negative control (Cont.). All results are expressed relative to WT K279a and are representative of three independent experiments. All measurements are means ± SEM from biological replicates. One-way-ANOVA followed by Tukey post hoc test for multiple comparisons; **** (*p* ≤ 0.0001), *** (*p* ≤ 0.001), ** (*p* ≤ 0.01), * (*p* ≤ 0.05), protease activity relative to WT K279a.

To elucidate the contribution of StmPR1 to total extracellular proteolytic activity of K279a, mutants were constructed in-house as described in Materials and Methods and similarly to reference [7]. To assay the activity of the K279a-ΔStr1: Erm mutants, 10 colonies were selected at random and protease activity measured by the Sensolyte Red Protease assay. All colonies selected had diminished protease activity (70.29 ± 0.94%) relative to the K279a wild (WT) type control. Interestingly, mutation of the StmPr1 gene incompletely abolished extracellular protease activity indicating a role of other proteases such as StmPR2 as previously described, or StmPR3 as described in this work (Figure 7) [7,8].

### 2.6. AAT, SLPI and Elafin Are Degraded in the Presence of K279a Culture Supernatant 

**Figure 8:**
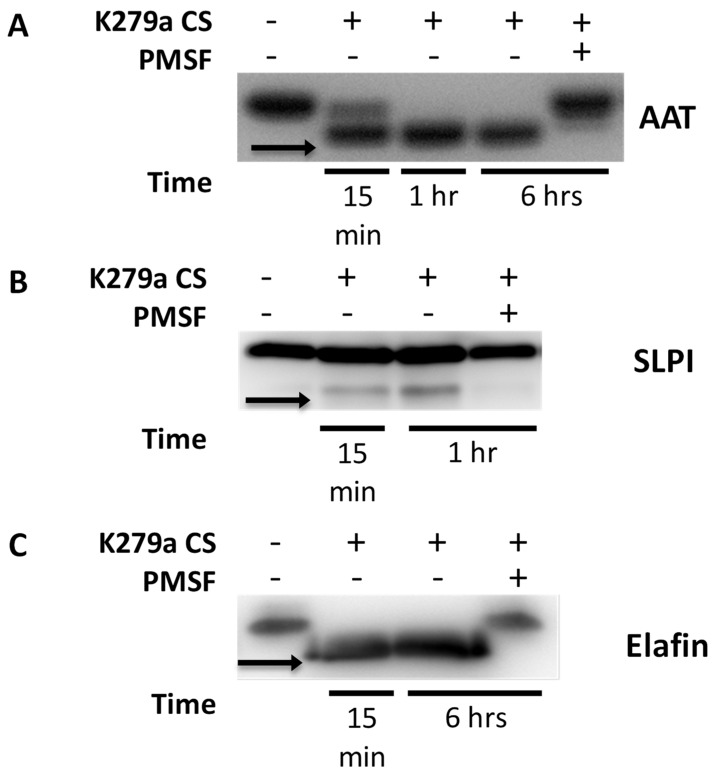
Effect of K279a culture supernatant on the integrity of the innate immune proteins: AAT, SLPI and Elafin. (A) AAT (0.83 μM), SLPI (0.83 μM) and elafin (0.42 μM) were incubated with K279a culture supernatant (CS) (5 × 10^3^ RFU/min) in the presence and absence of the protease inhibitor PMSF (1 mM) at 37 °C for various time points indicated above. Incubation products were analysed by Western under reducing conditions. Cleavage products of (**A**) AAT, (**B**) SLPI and (**C**) elafin are indicated by the arrows. Results are representative of three independent experiments.

Next, wild type K279a culture supernatant was used to evaluate the effect of proteases secreted by K279a on AAT, SLPI and elafin. As shown in Figure 8, AAT, SLPI and elafin were cleaved and/or degraded by K279a proteases after 15 min. Both AAT and elafin were completely degraded after 1 h. The serine protease inhibitor PMSF prevented cleavage, indicating that one or several *S. maltophilia* serine proteases were involved in this process. The ability of *S. maltophilia* proteases to cleave these proteins may render them incapable of inhibiting neutrophil elastase, the most abundant elastolytic enzyme in the CF lung. 

### 2.7. The Anti-NE Capacity of AAT, But Not SLPI or Elafin, Is Lost Due to K279a Proteases

**Figure 9:**
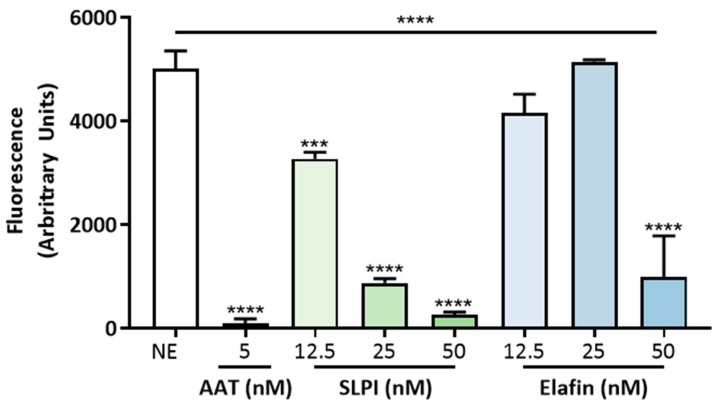
Inhibitory capacity of AAT, SLPI and elafin on neutrophil elastase activity. NE (5 nM) was used a positive control or incubated with an equimolar amount of intact AAT or increasing concentrations of SLPI (12.5, 25 and 50 nM) and elafin (12.5, 25 and 50 nM). NE activity was measured using a specific FRET substrate (Abz-Ala-Pro-Glu-Glu-IL-Met-Arg-Arg-Gln-EDDnp) in NE reaction buffer ((0.5 M NaCl, 0.1% (v/v) Brij-35 and 0.1 M HEPES, pH 7.5). Fluorescence intensity was measured at an emission wavelength of 420 nm after 120 s. All results are representative of three independent experiments. All measurements are means ± SEM from biological replicates. One-way ANOVA followed by Tukey post hoc test. *** *p* ≤ 0.001, **** *p* ≤ 0.0001, AAT, SLPI and elafin versus NE control.

Given that K279a proteases were capable of degrading AAT, SLPI and elafin, we investigated the consequences on their ability to inhibit neutrophil elastase (NE) using a specific fluorescence resonance energy transfer (FRET; Abz-Ala-Pro-Glu-Glu-IL-Met-Arg-Arg-GLn-EDDnp) substrate. Figure 9 demonstrates inhibition of NE activity by intact AAT, SLPI and elafin. AAT inhibits NE in a 1:1 ratio but a tenfold molar excess of SLPI and elafin was required to achieve significant levels of inhibition.

**Figure 10:**
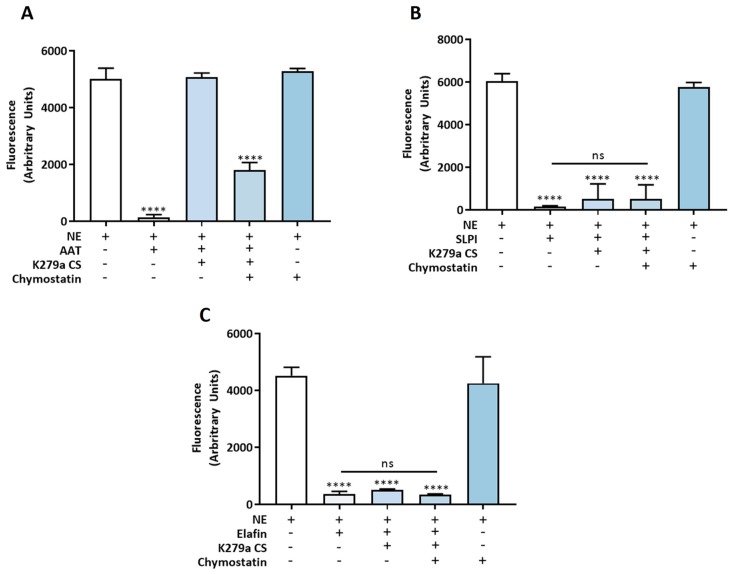
Effect of K279a proteases on the anti-NE capacity of AAT, SLPI and elafin. (**A**) Proteases on the anti-NE capacity of AAT. (**B**) Effect of K279a proteases on the anti-NE capacity of SLPI. (**C**) Effect of K279a proteases on the anti-NE capacity of elafin. NE (5 nM) was used as a positive control or incubated with an equimolar amount of intact AAT or a 10 fold molar excess of SLPI (50 nM) or elafin (50 nM) as a positive treatment control. AAT, SLPI or elafin were either untreated or treated with K279a culture supernatant in the presence or absence of chymostatin. NE (5 nM) and chymostatin (50 µM) represented the chymostatin control. NE activity was measured using a specific FRET substrate (Abz-Ala-Pro-Glu-Glu-IL-Met-Arg-Arg-Gln-EDDnp) in NE reaction buffer ((0.5 M NaCl, 0.1% (v/v) Brij-35 and 0.1 M HEPES, pH 7.5). Fluorescence intensity was measured at an emission wavelength of 420 nm after 120 s. All results are representative of three independent experiments. All measurements are means ± SEM from biological replicates. One-way ANOVA followed by Tukey post hoc test. **** *p* ≤ 0.0001, treatment versus NE positive control.

Next, AAT (5 nM), SLPI (50 nM) and elafin (50 nM) were left untreated or treated with K279a culture supernatant in the presence or absence of chymostatin. Chymostatin was used to inhibit K279a protease activity rather than PMSF in order to prevent any inhibitory effect of an exogenous protease inhibitor on NE activity, other than the three anti-proteases under investigation. Figure 10A shows that AAT lost its anti-NE capacity following treatment with K279a culture supernatant. This effect was abrogated in the presence of chymostatin indicating that a secreted protease(s) was responsible for this effect. Chymostatin had no inhibitory effect on NE, confirming its weak anti-NE activity and did not interfere with the NE inhibitory effect of AAT. Conversely, SLPI and elafin retained their ability to inhibit NE (Figure 10B,C) following treatment with K279a culture supernatant.

## 3. Discussion

Here we characterise the major extracellular proteases of *S. maltophilia* K279a. Previous studies have examined virulence determinants of *S. maltophilia* and although those did not directly determine which proteases were responsible for proteolytic activity, the data strongly indicated that StmPr1 is the major virulence factor of *S. maltophilia* [6,7,8,9]. Thus, while StmPR1 is the major reported contributor to extracellular protease activity, here screening and analysis of the K279a genome identified the previously described minor StmPR2 protease but also a relatively uncharacterised StmPR3 protease and a higher molecular weight extracellular serine protease (ExSP or Smlt4145) [8]. Using gelatin zymography, we identified four major bands and five minor bands of gelatinolytic activity. The activities of all major and three of the minor bands were inhibited in the presence of PMSF indicating they belong to the serine protease family of endopeptidases. Whether the four major bands represent four different proteases is difficult to ascertain directly from zymography. It is possible that one enzyme may be responsible for the presence of more than one band, being present in the form of a pro-enzyme (zymogen), being able to form dimers or undergoing degradation to truncated versions that are still active. While we can make inferences about the molecular weight (MW) of unknown proteases from zymography it must be borne in mind that accurate molecular weight determination can be hampered, given that some proteases exhibit slower migration through a gel that contains a substrate protein, meaning that an apparent protease may appear to have a greater MW than if run through a gel in the absence of substrate protein. 

In this work we undertook LC-MS/MS analysis of in-gel trypsin digests of bands excised from SDS-PAGE gels of K279a culture supernatant concentrate which approximated the major region of protease activity observed by zymography. Using this approach, we identified StmPR1, StmPR2, StmPR3, chitinase A and the outer membrane esterase. Spectral counting indicated that StmPR1 may not be the only major extracellular protease, but that StmPR3 may also be an important pathogenic protease in *S. maltophilia* infection. Concomitant with spectral counting from in-gel digests of identified proteases, StmPR3 was intermediate in LFQ intensity between StmPR1 and StmPR2, indicating that it may play an intermediary role between the major and minor extracellular proteases. 

We further examined the importance of StmPR1 as the major extracellular protease by creating a K279a mutant lacking *stmPr1*. The observed loss of protease activity in culture supernatants of StmPR1 mutants was estimated as 70% of the wild-type control, which highlighted the relative importance of other secreted proteases using a K279a model of *S. maltophilia* virulence. Studies to examine loss of the virulence by this mutant, or indeed double or triple StmPR mutants could prove interesting, but were beyond the scope of the current study. Neutrophil elastase is by far the most abundant (approximately 90%) serine protease in the lungs of persons with cystic fibrosis. High levels of proteinase-3 (PR3) [39] and cathepsins G [40], B, L and S [29] are also present in CF bronchoalveolar lavage fluid, as well as macrophage- and neutrophil-derived metalloelastases (MMP-8, MMP-9) [41] and elastolytic proteases expressed by *P. aeruginosa* [15]. Even though bacterial protease concentrations are small, their ability to cleave key anti-proteases that also exhibit anti-inflammatory, anti-microbial and immunomodulatory functions, underscores their potential role in the pathogenesis of the acquired mucosal immunodeficiency state that typifies CF lung disease. In the presence of *Pseudomonas* co-infection, although *Pseudomonas* proteases may be more abundant, rather than out-competing the effects of *S. maltophilia* proteases, the proteases from both species may collectively contribute to pathological effects. Furthermore, it is important to keep in mind that we characterised the expression pattern of *S. maltophilia* proteases at the peak of the growth phase in order to be able to study their biochemistry, whereas at suboptimal growth conditions such as may exist in the CF lung, some of these individual proteases might be more readily produced than others.

AAT is degraded by *S. maltophilia*, an effect which is reversed in the presence of the serine protease inhibitor PMSF. We have demonstrated that StmPR1 is the most abundant protease secreted by *S. maltophilia* K279a, indicating a potentially novel extracellular serine protease may be responsible for this effect. However, the other secreted serine proteases including StmPR3 and to a lesser degree StmPR2 may also be responsible. The cleavage and inactivation of AAT has important pathogenic consequence for cystic fibrosis. We have shown that AAT loses its anti-NE capacity following degradation by extracellular K279a proteases, an effect which is reversed by chymostatin. Depletion of AAT and an overabundance of NE can cause significant damage to the CF bronchial epithelium and act as a pro-inflammatory stimulus via activation of TLR4 through an EGFR pathway, amongst other effects [42].

SLPI was also cleaved in the presence of K279a proteases but that it was not completely degraded after six hours. While SLPI retained its ability to inhibit NE, SLPI levels are significantly decreased in the lungs of patients with cystic fibrosis secondary to degradation by NE and bacterial protease [1,35]. However, the ability of SLPI to retain its anti-NE capacity may have novel beneficial effects in the treatment of *S. maltophilia* lung disease if it can be shown that it can inhibit protease activity at higher concentrations (>0.83 μM) than the concentrations used in this study.

The role of the *S. maltophilia* extracellular serine proteases in the degradation of elafin was underlined in this study by the capacity of PMSF, a specific inhibitor of K279a serine protease(s) to prevent proteolytic degradation by K279a culture supernatant. Both *P. aeruginosa* PAO1-conditioned medium and two purified *Pseudomonas* metalloproteases, elastase and alkaline protease, have been shown to cleave recombinant elafin, but while elastase was shown to inactivate the anti-NE activity of elafin by cleaving its protease-binding loop, the antibacterial properties of elafin against PAO1 were found to be unaffected after elastase treatment [1]. In contrast to elastase, alkaline protease failed to inactivate the inhibitory properties of elafin against NE but cleaved elafin at the amino-terminal Lys6-Gly7 peptide bond, resulting in a decreased ability to covalently bind purified fibronectin following transglutaminase activity. Here, elafin retained its ability to inhibit NE following cleavage by *S. maltophilia* extracellular serine proteases, indicating that it can still function as a serine antiprotease in the *S. maltophilia* colonised lung. This is quite different from *Pseudomonas* proteases such as pseudolysin and aeruginolysin, which have diverse effects on the anti-NE and fibronectin binding qualities of elafin.

## 4. Conclusions 

The major secreted protease from K279a is a serine protease and its expression are culture condition dependent. Three serine proteases StmPR1, StmPR2 and StmPR3 were identified as the major, minor and intermediate proteases secreted by K279a in a DMEM *in vitro* model of *S. maltophilia* virulence, respectively. AAT is cleaved and inactivated by *S. maltophilia* proteases. SLPI and elafin are also cleaved by *S. maltophilia* but they retain their ability to inhibit neutrophil elastase. 

## 5. Materials and Methods 

### 5.1. Bacterial Growth and Reparation of K279a Culture Supernatant 

Fresh LBB was inoculated with 10 µL of *S. maltophilia* K279a (BIOMERIT Research Centre, Cork, Ireland) from an overnight culture that was grown overnight at 37 °C. To optimise the growth, medium required to induce protease production, 10 μL of overnight culture was incubated in 5 mL of LB broth (LBB), trypticase soy broth (TSB), M9 minimal medium or Dulbecco’s modified essential medium (DMEM, Invitrogen) low glucose (5.6 mM) or high glucose (25 mM) +/− 10% foetal calf serum (Invitrogen). To obtain K279a culture supernatant (CS) from cultures, they were centrifuged for five minutes at 1000 × *g* and the supernatant was decanted and filtered through a 0.22-μm-pore-size (Millex) filter to remove residual bacterial cells. 

### 5.2. Protease Activity Studies

Overnight cultures (100 μL) were transferred to agar wells created by means of a cork-borer (0.8 cm) in LB agar 2% (w/v) skimmed milk agar plates and incubated for 48 h at 37 °C. Plates were photographed after 24 and 48 h of incubation at 37 °C. Protease activity was indicated by a zone of clearing around the well. 

Protease activity in liquid culture supernatants was measured using the SensoLyte Red Protease Fluorometric Assay Kit (AnaSpec). In brief, 50 μL of a 1:200 dilution of protease substrate solution was mixed with 50 μL of test bacterial culture supernatant and the reaction incubated in the dark at 37 °C for 90 min. Culture medium or DPBS served as negative controls while 12.5 mU/μL of trypsin diluent (in H_2_O) was used as a positive control. Relative fluorescence intensity (RFU) was measured at Excitation/Emission (Ex/Em) = 544 nm/590 nm (Victor™ X3 Multilabel Plate Reader, PerkinElmer, Massachusetts, USA) and background fluorescence was removed by subtracting the readings from the substrate control (culture medium or H_2_O). 

### 5.3. PCR 

Bacterial DNA was isolated using the FastDNA Spin Kit (MP Bio) according to the manufacturer’s instructions. The extracted DNA was quantified on a NanoDrop 8000 (Thermo Scientific) and amplified by PCR using SYBR Green Master Mix (Roche, 04707516001) on a Light Cycler 480 (Roche). Primers used are listed in Table 3. PCR product samples were appropriately diluted in 6× DNA loading dye buffer (New England Biolabs) and loaded into separate wells of a 1.5% agarose gel in TAE buffer containing 1× SYBR Safe gel stain (Invitrogen). A 100 bp DNA ladder (New England Biolabs) was used as a marker for PCR product size. DNA was visualised under UV on a transilluminator and photographed using the Syngene G: BOX Chemi XL gel documentation system (Syngene, Cambridge, UK).

### 5.4. Gelatin Zymography 

A 12.5% SDS-polyacrylamide gel was prepared with the addition of gelatin (2 mg/mL) as the substrate. For estimation of protein size, 3 µL of pre-stained SeeBlue protein marker (Invitrogen) was loaded on each gel with a range spanning 4–250 kDa. After complete electrophoresis, the gel was placed in renaturing buffer [Triton X-100 2.5% v/v in developing buffer (50 mM Tris pH 7.6, 200 mM NaCl, 5 mM CaCl_2_ and 1 µM ZnCL_2_)] for 30 min at room temperature, followed by developing buffer overnight at 37 °C. The gel was stained with Coomassie Blue R250 (0.2% w/v) dissolved in 10% (v/v) acetic acid and 45% (v/v) methanol and then destained with 10% (v/v) acetic acid and 25% (v/v) methanol. 

### 5.5. Polyacrylamide Gel Electrophoresis and Western Immunoblotting

Culture supernatant and recombinant proteins (AAT (Athens Research), SLPI (R&D Systems), elafin (Proteo Biotech)) were quantified by bicinchoninic acid assay and electrophoresed on 12.5% SDS-polyacrylamide gels. For band size determination the protein marker SeeBlue® (Thermo Fisher Scientific: Waltham, MA, USA) Pre-stained Protein Standard (Invitrogen, LC5625) (Thermo Fisher Scientific: Waltham, MA, USA) or Mark12^TM^ Unstained Standard (Invitrogen, Bio Sciences Ltd, Ireland) was used. For Coomassie blue silver staining, gels were fixed in ethanol (50% v/v) and phosphoric acid (2%) for one hour and washed × 3 in dH2O prior to staining with Coomassie blue silver overnight. For western blotting, following SDS-PAGE, proteins were transferred to PVDF and signals were detected using goat anti-AAT (Abcam) (https://www.biocompare.com/), goat anti-SLPI (R&D Systems) or mouse anti-elafin (Abcam) with appropriate secondary antibodies and Immobilon Western chemiluminescent HRP substrate (Millipore) using the Syngene G:Box Chemi XL gel documentation system (Syngene International Limited, New Delhi, India). 

### 5.6. In-gel Digestion

Excised bands were destained with 100 mM ammonium bicarbonate/acetonitrile (1:1, vol/vol) for 30 min. Acetonitrile (500 μL) was added then removed, the gel pieces were dried and subjected to trypsin digestion overnight. Trypsin 20 μg/mL was prepared from proteomics grade Trypsin Singles (Sigma, T7575) (Sigma-Aldrich Chemicals company, St. Louis, MI, USA). Peptides were extracted into 100 μL 1:2 (vol/vol) 5% Formic acid/acetonitrile, vacuum dried and resuspended in 20 μL of sample preparation solution (0.5% trifluoracetic acid) for ZipTip Sample Preparation after which sample tubes were vacuum centrifuged and solvent was evaporated. Samples were resuspended in 20 μL of washing solution and frozen at −20°C prior to injection on LC/MS/MS. 

### 5.7. LC-MS/MS Analysis

The samples were run on a Thermo Scientific Q Exactive mass spectrometer connected to a Dionex Ultimate 3000 (RSLCnano) chromatography system. Tryptic peptides were resuspended in 0.1% formic acid. Each sample was loaded onto a fused silica emitter (75 μm ID, pulled using a laser puller (Sutter Instruments P2000)), packed with Reprocil Pur C18 (1.9 μm) reverse phase media and was separated by an increasing acetonitrile gradient over 47 min at a flow rate of 250 nL/min. The mass spectrometer was operated in positive ion mode with a capillary temperature of 320 °C, and with a potential of 2300V applied to the frit. All data was acquired with the mass spectrometer operating in automatic data dependent switching mode. A high resolution (70,000) MS scan (300–1600 m/z) was performed using the Q Exactive^TM^ (Thermo Fisher Scientific: Waltham, MA, USA) to select the 12 most intense ions prior to MS/MS analysis using HCD (high-energy-C-trap dissociation). 

### 5.8. Protein Identification and Peptidomic Data Analysis

Raw data from the Orbitrap Q-Exactive was processed using MaxQuant version 1.5.0.30 [43,44] incorporating the Andromeda search engine [45]. To identify peptides and proteins, MS/MS spectra were matched to the Uniprot database of *S. maltophilia* (release 2014_01 containing 49,886 entries). All searches were performed in specific trypsin digestion mode. The database searches were performed with cysteine carbamidomethylation as fixed modification and acetylation (protein N terminus) and oxidation (M) as variable modifications. Mass spectra were searched using the default setting of MaxQuant namely a false discovery rate of 1% on the peptide and protein level. For the generation of label free quantitative (LFQ) ion intensities for protein profiles, signals of corresponding peptides in different nano-HPLC MS/MS runs were matched by MaxQuant in a maximum time window of 0.7 min [46].

The Perseus statistical software (version 1.5.3.2) (https://maxquant.net/perseus/) [47] was used to analyse the protein LFQ intensities.

### 5.9. Construction of K279a StmPR1 Knock-Out

Site directed inactivation of the gene (smlt0686) in K279a was done by *sacB* counter selection with the suicide vector pEX18Tc [48]. To generate a plasmid construct containing StmPR1, the ORF of the smlt0686 (StmPR1) was amplified using K279a genomic DNA as a template using the primer pair Str1-For and Str1-Rev (Table 1). The amplified fragment was double digested with *Sac*I and *Xba*I and cloned into their respective restriction sites in pEX18Tc, creating pEX18Tc-Str1. This was verified by sequencing. This vector was heat-shock transformed into competent NEB 5-α *E. coli* cells and was plated onto LB plates supplemented with 10 μg/mL tetracycline (Tc). Using the purified pEX18Tc-Str1 plasmid as a template, a vector containing an 846 bp deletion in StmPR1 (pEX18Tc-ΔStr1) was constructed by inverse PCR using the primers Str1Inv-5’ and Str1Inv-3’ (Table 1). This also introduced a unique *Bam*H1 site. Next, an erythromycin (Erm) cassette was amplified from the pGEM-Erm plasmid [49] with primers PErm5′ and PErm3′ (Table 1). Subsequently, the *Bam*H1 digested PCR product was ligated into the *Bam*H1 site of pEX18Tc-ΔStr1 to yield pEX18Tc-ΔStr1:: Erm which carries smlt0686 interrupted by with Erm resistance gene. Plasmid pEX18Tc-ΔStr1::Erm was verified by sequencing. Electrocompetent K279a cells were transformed with pEX18Tc-ΔStr1::Erm. Mero-diploid colonies were obtained by selection on LB agar containing Tc (17 μg/mL) and Erm (64 μg/mL). Finally, potential double-cross over mutants with an Erm^r^-Suc^r^ phenotype were selected on LB agar + 5% w/v Sucrose (without NaCl) and Erm 500 μg/mL. Mutants were verified by PCR. 

### 5.10. Neutrophil Elastase Inhibition Studies

NE inhibition studies were performed using 5 nM of NE (reconstituted in 0.05 M sodium acetate, 0.1 M NaCl, pH 5.0) with an equimolar amount of untreated (control) AAT or a 10-fold molar excess of SLPI (50 nM) or elafin (50 nM). To study the effect of K279a culture supernatant on AAT, SLPI and elafin, each protein was incubated in DPBS for 1 h at 37 °C and were either treated with K279a culture supernatant (400 RFU/μg) or treated with K279a culture supernatant which had been inhibited by chymostatin (50 µM) for 15 min. Activity of NE was measured using a specific fluorescence resonance energy transfer (FRET; Abz-Ala-Pro-Glu-Glu-IL-Met-Arg-Arg-Gln-EDDnp) substrate [50] in NE reaction buffer (0.5 M NaCl, 0.1% (v/v) Brij-35 and 0.1 M HEPES, pH 7.5). Fluorescence intensity was measured in a 96 well black polystyrene flat bottomed microplate at an emission wavelength of 420 nm on Victor™ X3 Multilabel Plate Reader (PerkinElmer, MA, USA) for 120 s. Reactions involving the FRET substrate were carried out in the dark. Background fluorescence was removed by subtracting the readings from the substrate control. 

### 5.11. Statistical Analysis 

All statistical analyses were performed using GraphPad Prism 5.0 software package (San Diego, CA). All experiments were performed in triplicate and results are expressed as the mean ± SEM and were compared by One-way-ANOVA followed by Tukey post hoc test for multiple comparisons. Differences were considered significant at *p* ≤ 0.05.

## Figures and Tables

**Table 1:** Characteristics of candidate K279a extracellular serine proteases.

	StmPR1	StmPR2	StmPR3	ExSP
UniProt ID	B2FNH2	B2FQ06	B2FLH5	B2FI22
Gene	Smlt0686	expR	Smlt4395	Smlt4145
Length (AA)	630	580	588	1,118
Predicted MW	63,605 Da	58,291 Da	61,297 Da	116,012 Da
Predicted PI	6.47	6.22	9.14	6.15
MEROPS family	S8 (subtilisin)	S8 (subtilisin)	S8 (subtilisin)	S8 (subtilisin)

Abbreviations: AA, amino acid; MW, molecular weight; PI = isoelectric point.

**Table 2:** Proteases identified from in gel-digestion of bands 3-6 from K279a culture supernatant.

	Spectral Count	% Coverage	MW (Da)
StmPR1	15	37.3	63,605
StmPR2	2	8	58,291
StmPR3	16	38.1	61,297
Chitinase	1	1.7	72,939
Esterase	11	27	64,199

**Table 3:** Primers used in this study.

Primer	Description	Sequence (5’–3’)	Length (bp)	GC (%)	Tm (°C)	Product (bp)
16s rRNA-F	16s ribosomal RNA	CAGCTCGTGTCGTGAGATGT	20	55	54	193
16s rRNA-R		AGCCCTCTGTCCCTACCATT	20	55	54	
FtsH-F	ATP dependent metalloproteinase	GTGGCAACGAGAAGGAAGTC	20	55	54	179
FtsH-R		CTTGTAGACCGGGTCATGCT	20	55	54	
StmPR1-F	StmPR1 protease	CAACGACTCGATGAATGTGG	20	50	52	174
StmPR1-R		CAGACATAGCCGTTCGGATT	20	50	52	
StmPR2-F	StmPR2 protease	CAGGTCGAGAGCATCATCAA	20	50	52	168
StmPR2-R		GGTCACCGGTACGTTGTTCT	20	55	54	
StmPR3-F	StmPR3 protease	AGCGAAAACACGATTCGTTC	20	45	50	189
StmPR3-R		ACGGTGATGACGTTGAACAG	20	50	52	
ZnMMP-F	Zinc metalloproteinase	CGGTCGGTGTAGAAGTTGGT	20	55	54	175
ZnMMP-R		ACTACGCCGCTTACATGGAC	20	55	54	
Esterase-F	Esterase	TTAGAAGGTGCCGCTGAAGT	20	50	52	152
Esterase-R		GCCTGAAGTTCGACAAGGAC	20	55	54	
ExSP-For	Extracellular serine protease	TGGTGAAACCGACTACGTGA	20	50	52	181
ExSP-Rev		GTACGTGGGATTCTGGCTGT	20	55	54	
Str1-For	StmPR1 gene cloning	GTCGAGCTCGTGATCAAGAAGCAGAAC	27	52	62	
Str1-Rev		CAGTCTAGATTACTGCGTGGCGAGAATG	28	52	58	
Str1Inv-5’	StmPR1 gene deletion (inverse PCR)	CTAGGATCCGTACGGATCGTTGGGCAC	27	59	57	
Str1Inv-3’		ATAGGATCCGCGCCACATGTGGCTGCC	27	63	63	
PErm5′	Erythromycin cassette	GACGGATCCGAAACGTAAAAGAAGTTATG	29	41	59	
PErm3′		GTCGGATCCTACAAATTCCCCGTAGGC	27	56	64	
